# Palliative care for cancer patients in a primary health care setting: Bereaved relatives' experience, a qualitative group interview study

**DOI:** 10.1186/1472-684X-7-1

**Published:** 2008-01-15

**Authors:** Mette Asbjoern Neergaard, Frede Olesen, Anders Bonde Jensen, Jens Sondergaard

**Affiliations:** 1 The Research Unit for General Practice, University of Aarhus, Denmark; 2 Department of Oncology, Aarhus University Hospital, Denmark

## Abstract

**Background:**

Knowledge about the quality and organisation of care to terminally ill cancer patients with a relatives' view in a primary health care setting is limited.

The aim of the study is to analyse experiences and preferences of bereaved relatives to terminally ill cancer patients in a primary care setting to explore barriers and facilitators for delivery of good palliative home care.

**Methods:**

Three focus group interviews with fourteen bereaved relatives in Aarhus County, Denmark.

**Results:**

Three main categories of experience were identified: 1) The health professionals' management, where a need to optimize was found. 2) Shared care, which was lacking. 3) The relatives' role, which needs an extra focus.

**Conclusion:**

Relatives experience insufficient palliative care mainly due to organizational and cultural problems among professionals. Palliative care in primary care in general needs improvement and attention should be drawn to the "professionalization" of the relatives and the need to strike a balance between their needs, wishes and resources in end-of-life care and bereavement.

## Background

A palliative course of disease often involves several different parts of the healthcare system, and the collaboration or shared care therefore becomes an important issue (Table 1)[1].

**Table 1:** Definition and implications of Shared care

Shared care	«Shared care is the joint participation of general practitioners and hospital consultants in the planned delivery of care for patients with a chronic condition, informed by an enhanced information exchange over and above routine discharge and referral letters» [29]
	Shared care demands knowledge of the abilities and the qualities of the cooperating partners, but also accept of each others' roles in the delivery of palliative care [1].

One group of health care professionals often involved is the primary care professionals, e.g. the general practitioners (GPs).

GPs are expected by their patients, policy makers and health organisations to be involved in palliative home care [2,3]. Many GPs consider palliative care to be typical GP work and one of the best parts of the job [4]. The traditions and values of general medicine corroborate the intentions of palliative care [5,6], e.g. in seeking a continuing relationship between patient, family and professionals, the continuity of care [7] and the ability to perform care in co-operation with other health professionals [8].

GPs' management of palliative care, especially symptom control, has often been a subject of debate. It has been suggested that poor delivery of care in the primary sector may be a major reason why the majority of cancer patients die in hospitals despite their and their relatives' preference for a home-death [9-11]. Hence, there is a profound need for a deeper insight into barriers and facilitators for delivery of good palliative home care.

Evaluation of palliative care has often focused on clinical measures, e.g. the professionals' ability to provide symptom control, at the expense of the cancer patient's and relatives' perception of the total care delivery in the terminal phase. However, the bereaved relatives have invaluable experience of primary health care delivery from the terminal diagnosis until bereavement.

We therefore aimed to analyse experiences and preferences of bereaved relatives to terminally ill cancer patients in a primary care setting to explore barriers and facilitators for delivery of good palliative home care.

## Methods

We conducted three qualitative, semi-structured group interviews with fourteen relatives of recently deceased cancer patients in Aarhus County (640 000 inhabitants), Denmark (5.5 million inhabitants) from June to October 2005.

### Setting

The Danish Health care system is financed through taxes and provides free and equal access to health care services. More than 98% of the Danes are registered with a GP and receive free medical care [12]. Danish GPs provide most of the health care themselves and act as gatekeepers for access to specialist treatment.

Specialist palliative teams with consultants, nurses and other healthcare providers are attached to the three major hospitals in the County of Aarhus, and the GPs can refer patients to these teams or ask the specialists for advice. These teams are only available during normal daytime hours.

Palliative home care is divided into a basic and a specialist level [13]. The basic level professionals are the primary care sector, e.g. the GPs, the home care nurses and home care services. The professionals involved in specialist level home care are the primary care sector plus a palliative specialist team, working either as consultants or as active professionals in the patient's home.

At the moment there is no formalised education in palliative care of significance at the pre-graduate level of doctors in Denmark. At the level of specialising toward General Medicine there is a one-day course of theoretical aspects of palliative care. The Danish GPs can participate in non-compulsory courses in palliative care, but these courses are in competition with courses in all other aspects concerning general medicine.

### Sample

The informants were close relatives of recently deceased cancer patients, and the inclusion criteria can be seen in Table 2. The criteria that the patient died less than one year before the inclusion of the relatives was used to minimise memory decay.

**Table 2:** Inclusion criteria

Inclusion criteria for bereaved relatives	The relative must be closely related to an adult deceased cancer patient
	The relative must have been involved in the palliative care of the deceased patient
	The cancer patient must have died less than one year before the inclusion
	The relative must be 18 years or more
	The relative must speak and understand Danish
	The relative must be mentally and physically able to participate in a focus group interview

The home care services in the 26 municipalities of Aarhus County and the specialist palliative team at Aarhus University Hospital were asked to identify possible informants meeting the inclusion criteria. The specialist palliative team identified two possible informants who were both invited and accepted to participate. The other 16 possible informants were identified by the home care services. In all, we invited 18 relatives of whom 14 agreed to be interviewed (78%). Four relatives were not able to participate on the proposed dates, which gave us the problem that only three informants participated in the first interview. However this did not influence on the interaction in the groups that were just as spirited in all three groups. Hence, the three interview groups comprised three, five and six informants, respectively (Table 3). We ensured a wide range of demographic characteristics and in time since death of the patient (Table 4) [14]. One participant (nr. 13) were only one month past bereavement. This relative had specifically stated to the home care service that he wanted to help others in the same situation if possible.

**Table 3:** The informants

**Nr of Informants**	**Group interview number**	**Relation to deceased**	**Gender**	**Age of relative**	**Age of deceased**	**Type of cancer**	**Place of death**	**Months from death to interview**
1	1	Daughter	Female	40	64	Pancreas	Daughter's home	5
2	1	Spouse	Female	55	60	Kidney	Home	5
3	1	Sister	Female	61	63	Mamma	Hospice	2
4	2	Spouse	Female	75	76	Urinary	Home	9
5	2	Spouse	Male	60	56	Ovarian	Hospital	6
6	2	Spouse	Female	46	56	Pleural	Home	9
7	2	Sister	Female	40	60	Brain	Hospice	15
8	2	Spouse	Female	73	83	Prostate	Nursing home	10
9	3	Daughter	Female	40	72	Lung	Daughter's home	6
10	3	Spouse	Male	75	65	Lung	Home	16
11	3	Spouse	Female	70	78	Lung	Hospital	3
12	3	Daughter	Female	44	78	Lung	Hospital	3
13	3	Spouse	Male	45	44	Colon	Home	1
14	3	Daughter	Female	49	83	Malignant melanoma	Nursing home	7

**Table 4:** Demographics

Deceased cancer patients: (14 patients (100%))	Sex	Women	7 patients (50%)
		Men	7 patients (50%)
	Age at time of death, median (range)		64.5 years (44; 83)
	Patients in contact with a specialist palliative care team		11 patients (79%)
Place of death:	Home		7 patients (50%)
(14 patients (100%))	Hospital		3 patients (21%)
	Hospice		2 patients (14%)
	Short-term nursing home		2 patients (14%)

Time from patient's death to interview with relative, median (range)			6 months (1; 16)

Relatives interviewed (14 informants (100%))	Sex	Women	11 informants (79%)
		Men	3 informants (21%)
	Family relationship to patient	Spouse	8 informants (57%)
		Child	4 informants (29%)
		Sibling	2 informants (14%)
	Age at interview, median (range)		52 years (40; 75)
	Place of residence	Urban	3 informants (21%)
		Semi-urban	5 informants (36%)
		Rural	6 informants (43%)

### Group interviews

All group interviews were conducted at The Research Unit for General Practice, The University of Aarhus. The group interviews were conducted by MAN, who was supervised by the co-authors, JS or FO. All three are specialists in general practice and JS and FO are GPs. MAN has completed courses in interview technique and analysis, and JS and FO are experienced in qualitative research methods.

The group interviews, which were tape-recorded with the informants' consent, were guided by a topic guide based on clinical experiences and literature studies (Table 5). The topic guide covered the following main areas: The health professionals' management, including the role and management of the GP, the organisation, interpersonal relation and co-operation in palliative care [15]. The guide was further developed according to the themes emerging during the analyses conducted after each group interview, e.g. after the first group interview the role of the relatives was added to the topic guide. No new themes were added after the two following interviews. Open-ended questions were used. The informants were encouraged to speak freely and to raise issues of importance to them. The group interviews lasted from 111 to 129 minutes. A summary was given at the end of each group interview to obtain an immediate validation of the themes identified by the researcher.

**Table 5:** Topic guide themes

Themes	Issues	Examples of questions
The health professionals' management	The role and management of the GP	How where the management of the GP involved? What was good/bad? How could it be improved? Which role did you wish the GP had played? (Following aspects were elaborated about the GP: knowledge, resources, availability, continuity, barriers)
	Discharge from hospital care to home care	How was the quality of the discharge from the hospital? How was the co-operation in this situation among the sectors? What was good/bad? How could it be improved?
	Interpersonal relation	How was the co-operation among the GP and the home care nurses/services? How was the co-operation among the GP and the hospital/palliative specialist teams during the course of disease at home? What was good/bad? How could it be improved?
	Organisation of palliative care	How do you think palliative care should be organised?

The role of the relatives		Did you get enough support from the professionals involved as a relative? What was good/bad? How could it be improved?

### Analysis

The group interviews were transcribed verbatim by a trained professional secretary. All transcripts were read simultaneously with the sound of the tapes by MAN to ensure correctness of the transcription and were read repeatedly by the authors to get an overall impression of the material before the initial coding [16]. A qualitative description approach was used for the analysis[17]. Following this analytic approach we enhanced rigour by focusing on the following strategies: 1) authenticity, the attention to the voices of participants and the ability to remain true to the phenomena under study, 2) credibility, a reflexion of how believable results are, 3) criticality, the critical appraisal of every decision made throughout the research process and 4) integrity, demonstrated by on-going reflection and self-criticality of the researcher [18].

All meaningful text units were identified and coded. The codes were subsequently grouped into relevant categories by MAN and at the end seven categories were identified, which again were grouped into three main categories. In this way we allowed the main categories to evolve from the data instead of imposing a framework *a priori *[19]. No new categories emerged from the analysis of the third group interview.

Agreement was reached in the group of authors after thorough discussion of the initial coding, the categories and the main categories. We used the software package *NVivo, ed. 6 *(QRS international, Melbourne, Australia) to assist in the coding, sorting and retrieval of data.

## Results

**Figure 1:**
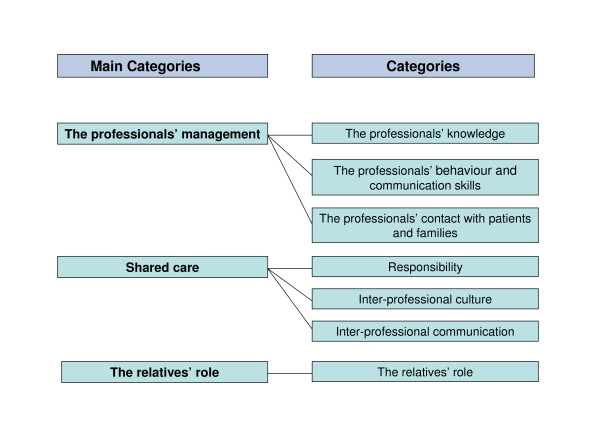
Final categories.

Seven categories were identified and grouped into three main categories: the health professionals' management, shared care and the relatives' role (Figure 1).

### The health professionals' management

The analyses revealed that the relatives found three aspects of the professionals' management of palliative home-care to be important: The professionals' knowledge, their behaviour and communication skills and their contact with patient and families.

#### The health professionals' knowledge

Some relatives experienced that their GP was proficient in handling symptom control and other aspects of the treatment of terminally ill cancer patients.

*"As soon as we were there, the GP looked things up on the computer and she knew all the time what was going on... she really did." *(Informant 10)

Others felt that the GPs neither possessed sufficient knowledge nor sought assistance from the palliative care specialists.

*"... when the GP cannot manage, he should refer to someone who can. Our GP is the kind of person who thinks he knows best." *(Informant 2)

#### The health professionals' behaviour and communication skills

Empathy and time to listen were important qualities to the relatives.

*"... a good thing about my GPs is ... both the female and the male GP in my practice are very competent and have the skills necessary to talk to the patients. They have a way with people." *(Informant 1)

Some of the doctors, nurses and other health-care providers were able to communicate competently with the patients and their relatives, while others did not seem to possess these skills.

"And as I told you before, our own GP actually referred to me as "the widow" already in advance. I think that was a very improper remark." (Informant 2)

"They must have a flair for breaking bad news. Some doctors think you have to know everything, whether you want it or not" (Informant 6)

#### The professionals' contact with patients and families

The relatives requested that health professionals be more active in establishing personal contact with the family.

*"... perhaps the GP could do a little outreach work and phone us asking how we were doing ... the GP did not have the slightest idea of what was going on." *(Informant 9)

The relatives described how hard it was always to be the one to take initiative.

*"I think it has been very hard work the way we constantly having to keep abreast." *(Informant 12)

Some GPs visited the patients at home on their own initiative, whereas others refused to make home visits at all, e.g. because they lacked time. GPs' home visits were highly appreciated because of the profound feeling of security it gave the patient and relatives.

*"The GP spontaneously came to visit us every day until it was over. He came and asked if there was anything he could do. And it was an incredible relief not having to call him first... Then, I really felt that he gave me all the support a doctor could give."*(Informant 13)

The informants found it important to know whom to contact during the out-of-hours periods, e.g. evenings, nights, weekends and national holidays. In some cases, the GP gave the family his or her private telephone number for such situations, which was highly appreciated, while others had to use the on-call GPs who were unfamiliar with the patient. The informants also appreciated whenever the health care professionals made an active contact after bereavement, because they often felt left in limbo.

### Shared care

Three categories emerging from the analyses addressed shared care between the different professionals involved: Professional responsibility, inter-professional culture and inter-professional communication

#### Professional responsibility

Often, the GPs were not even informed about the patients' discharge from hospital to palliative care at home since they received the discharge letters very late. This failure to pass on responsibility made patients and relatives feel as though they were "left in limbo".

*"You see, at first, when we were told by the hospital physician that there was nothing more to do but control the pain, at that time, I felt left alone... ... We were simply left in limbo." *(Informant 2)

During the palliative course of disease in the patient's home, the relatives wanted one of the health-care professionals to take on the role of team-leader, i.e. to co-ordinate care and take control of the situation, and they preferred this professional to be the GP. They appreciated the continuity the GP provided, i.e. the GP's acquaintance with the patient and the relatives both before and after the cancer diagnosis and the GP's knowledge of the specific course of disease.

*"Well, I think the GP should have the opportunity to be in charge of the coordination..." *(Informant 5)

#### Inter-professional culture

In many cases the relatives experienced a lack of respect between the professionals especially between primary health care professionals and hospital professionals.

*"... it was not a comforting message we got, that our GP had referred my husband to the hospital too late ... they told us that straight out. A hospital physician told us that they always get the patients too late from that municipality." *(Informant 11)

The insinuation of other professionals' incompetence gave the relatives a feeling of insecurity and they did not know whom to trust.

#### Inter-professional communication

Many relatives experienced communication problems between the hospital and the primary care sector.

*"Honestly, I sometimes think that there is a wide gap between the GPs and the hospitals ... Well, I wish they had closer contact." *(Informant 1)

Furthermore, the relatives often experienced that the GPs and the home care nurses did not receive the necessary information from the hospitals. The relatives did not understand why GP and hospital records were not accessible on-line to all the professionals involved.

*"Our GP asked me to call him, when I got news from the hospital, because it often took too long before he got the information himself..." *(Informant 6)

Poor communication between GPs and home care nurses was perceived as a major problem. In some cases the relatives experienced that the nurses were unable to reach the GP by phone, and the relatives saw the home care nurses' frustration.

*"But I know that the home nurses were very frustrated because they couldn't contact our GP." *(Informant 14)

### The role of the relatives

All relatives stated that before the patient's death both they and the patients wished that end of life care and death should take place at home. Despite that, only half died at home (Table 4).

All relatives stated that they were grateful to have had the opportunity to participate in the care of their terminally ill relatives.

"They should be better at explaining people what it actually means to take your loved ones home and care for them there. How much help you can actually get ... And it is not dangerous because the professionals are there for you, all of them."

(Informant 12)

However, an experience shared among the relatives was an unspoken pressure from the professionals to be "semi-professionals" themselves rather than just relatives, i.e. a "professionalization" of the relatives took place.

*"My husband's family noticed that when I was with my husband, I did not act as his wife, but as a professional when it came to care and when the GP visited..." *(Informant 6)

*"I insisted on being allowed to be just a relative. And that was difficult although I told them I had a right to that." *(Informant 14)

The pressure of being a "semi-professional" was especially a problem when the relatives happened to be health care professionals themselves, when the relatives had to provide intimate care for the patient and when the patient needed 24-hour care.

*"... you have to be available twenty-four hours a day. My eighty-year-old dad cried at night when it became too much for him taking care of my terminally ill mother. "I cannot do this anymore", he cried. And then I had to take over..." *(Informant 14)

In one case the relative stated that the burden of the role of a "semi-professional" meant that the family gave up on the possibility of home death. This left them with a feeling of inadequacy as relatives since they could not fulfil the patient's wish for a home death.

## Discussion

Since the relatives felt an unspoken pressure from the professionals to be "semi-professionals" themselves and a "professionalization" of the relatives took place, the relatives asked for health professionals who had the necessary knowledge, who were readily available to the patients and their families and more active in establishing personal contact with them. They also described both organizational and cultural problems in the health care system.

Focus group interviews are pertinent, as individual interview to explore the informants' perspectives and experiences concerning a specific topic. The force of group interview is that the interaction in the group can be utilised as a factor of development of views and description among the participants and can make new themes or aspects of the topic emerge [20]. In this way one gets a broad elaboration of the topic. In our study the group setting made new themes emerge but it also gave the relatives a valuable feeling of shared experience and of being able to support each other in the interview situation.

Interviews with terminally ill patients could have added important information to our study, but arranging interviews with terminally ill patients would have entailed ethical and practical difficulties disproportionate to the added value. Choosing close relatives who had participated in the palliative care as informants gave us an insight into the patients experience as well and it gave us the opportunity to explore the role of the relatives.

There was a surprising polarization in the experiences of GPs' management of palliative care. However, most experiences were negative in nature. This may be due to the fact that the relatives who agreed to participate in the group interview were the ones who needed to express their bad experiences which the authors feel that there is a tendency towards in the Danish society or of course might be due to an actual bad performance of the GPs involved. However, this can not be determined in a qualitative study, and will require a follow-up with a quantitative study.

The fact that the interviewers (MAN, JS, and FO) were all specialists in general practice could bias the group interviews and the analyses, but all authors were aware of this potential problem [14].

In earlier studies of the professionals' management of palliative care some relatives were not satisfied with symptom control in primary care, but they still rated the overall GP care as very good [10]. The overall satisfaction with the GPs was significantly associated with symptom control, time to listen and accessibility [21]. GP home-visits may increase terminally ill cancer patients' possibility of dying at home [22]. In our study, continuity of care and good knowledge of the whole course of disease were among the reasons why the GPs were valued despite their at times poor management of palliative care.

Most studies examining the relatives' role have described that the relatives become emotionally and physically exhausted [11,23]. We found that an important reason for this could be their "professionalization" and the absence of support from health care professionals, particularly after bereavement. It is important that the professionals help the relatives strike a balance between their needs, wishes and resources [23,24]. This balance can be difficult to find in end-of-life care situations, where the relatives feel obliged to fulfil the wishes of the dying relative.

The importance of communication between GPs and specialists and the request for shared care have also been found in other palliative studies [25-28]. Also in line with our findings, these studies emphasize that continuous contact with the GP can be maintained by referring the patients back to their GPs after hospitalization, by ensuring good communication between specialists and GPs and by providing a clear definition of the GP's role [26]. However, a fundamental prerequisite for successful shared care is the presence a coordinator [25]. The need for and lack of shared care is well-described, but the reasons for this lack of shared care and ways of improving it are less well-explored. From our study it appears that one of the main barriers to optimizing shared care may be the lack of a shared care culture among health professionals and the lack of a home-care team leader in palliative care.

The issue of "professionalization" of the relatives was an important finding of our study and the extend and significance of this aspect should be further elaborated in future research. It is a challenge to all professionals involved in palliative home care to help relatives to strike a balance between the needs, wishes and resources f the relatives.

Another main challenge to the health care system is to create a shared care culture characterised by cooperation and mutual respect among professionals within the health care system. Further research in this subject is in place, but it seems that the respect and knowledge among different types of professionals are to some extend missing, and maybe shared care is missing as a focus already in the education and training of doctors, nurses and other professionals involved.

Hence, in order to optimize palliative home care, further research is required into how to develop a shared care culture, how to improve communication along the cancer journey, how to delegate professional responsibility and on relatives role.

A questionnaire survey among relatives, home care nurses and GPs is currently being conducted in order to give quantitative estimates of the distributions of opinions and experiences.

## Conclusion

Our study indicates that relatives experience insufficient palliative care, mainly due to organizational and cultural problems among professionals. There is a lack of shared care. Palliative care in primary care in general needs improvement, both in terms of professional knowledge, attitude, availability and communication skills. Furthermore, attention should be drawn to the "professionalization" of the relatives and the need to strike a balance between their needs, wishes and resources in end-of-life care and bereavement.

## Competing interests

The author(s) declare that they have no competing interests.

## Authors' contributions

MAN participated in the design of the study, conducted the interviews and the analysis and drafted the manuscript.

FO and JS participated in the design of the study, as supervisors in conducting the interviews and participated in discussions of categories.

ABJ participated in the design of the study.

All authors read and approved the final manuscript.

## Pre-publication history

The pre-publication history for this paper can be accessed here:



## References

[B1] Bliss J, Cowley S, A W (2000). Interprofessional working in palliative care in the community: a review of the literature. Journal of interprofessional care.

[B2] Department of Health (2000). The NHS Cancer Plan. A plan for investment, A plan for reform.

[B3] The Danish National Board of Health, Kræftstyregruppen (2005). [National Danish Cancer Action Plan] National kræftplan 2.

[B4] Groot MM, Vernooij-Dassen MJ, Crul BJ, Grol RP (2005). General practitioners (GPs) and palliative care: perceived tasks and barriers in daily practice. Palliat Med.

[B5] Allen J, Gay B, Crebolder H, Heyrman J, Svab I, Ram P (2002). The European Definitions of the Key Features of the Discipline of General Practice: the role of the GP and core competencies. Brit J Gen Pract.

[B6] (1990). Cancer Pain Relief and Palliative Care Report of a WHO Expert Committee.

[B7] von B, Eliasson G, Sarvimaki A, Mattsson B, Hjortdahl P (2006). Patients' views on interpersonal continuity in primary care: a sense of security based on four core foundations. Fam Pract.

[B8] Nielsen JD, Palshof T, Mainz J, Jensen AB, Olesen F (2003). Randomised controlled trial of a shared care programme for newly referred cancer patients: bridging the gap between general practice and hospital. Qual Saf Health Care.

[B9] Thomas C (2005). The place of death of cancer patients: can qualitative data add to known factors?. Soc Sci Med.

[B10] Hanratty B (2000). Palliative care provided by GPs: the carer's viewpoint. Br J Gen Pract.

[B11] Brazil K, Howell D, Bedard M, Krueger P, Heidebrecht C (2005). Preferences for place of care and place of death among informal caregivers of the terminally ill. Palliat Med.

[B12] de Fine Olivarius N, Hollnagel H, Krasnik A, Pedersen PA, Thorsen H (1997). The Danish National Health Service Register. Dan Med Bull.

[B13] The National council for palliative care (2007). Palliative care explained. http://www.ncpc.org.uk/palliative_care.html.

[B14] Crabtree BF and Miller WL (1992). Doing qualitative research.

[B15] Donabedian A (1978). The quality of medical care. Science.

[B16] Giorgi A, Giorgi A (1985). Sketch of a psychological phenomenological method. Phenomenology and psychological research.

[B17] Sandelowski M (2000). Whatever happened to qualitative description?. Res Nurs Health.

[B18] Milne J, Oberle K (2005). Enhancing rigor in qualitative description: a case study. J Wound Ostomy Continence Nurs.

[B19] Pope C, Ziebland S, Mays N Qualitative research in health care. Analysing qualitative data. BMJ.

[B20] Kitzinger J (1995). Qualitative research. Introducing focus groups. BMJ.

[B21] Lecouturier J, Jacoby A, Bradshaw C, Lovel T, Eccles M (1999). Lay carers' satisfaction with community palliative care: results of a postal survey. South Tyneside MAAG Palliative Care Study Group. Palliat Med.

[B22] Aabom B, Kragstrup J, Vondeling H, Bakketeig LS, Stovring H (2006). Does persistent involvement by the GP improve palliative care at home for end-stage cancer patients?. Palliat Med.

[B23] Grbich C, Parker D, Maddocks I (2001). The emotions and coping strategies of caregivers of family members with a terminal cancer. J Palliat Care.

[B24] Proot IM, Abu-Saad HH, Crebolder HF, Goldsteen M, Luker KA, Widdershoven GA (2003). Vulnerability of family caregivers in terminal palliative care at home; balancing between burden and capacity. Scand J Caring Sci.

[B25] Steinhauser KE, Christakis NA, Clipp EC, McNeilly M, McIntyre L, Tulsky JA (2000). Factors considered important at the end of life by patients, family, physicians, and other care providers. JAMA.

[B26] Norman A, Sisler J, Hack T, Harlos M (2001). Family physicians and cancer care. Palliative care patients' perspectives. Can Fam Physician.

[B27] Evans R, Stone D, Elwyn G (2003). Organizing palliative care for rural populations: a systematic review of the evidence. Fam Pract.

[B28] Borgsteede SD, Graafland-Riedstra C, Deliens L, Francke AL, van Eijk JT, Willems DL (2006). Good end-of-life care according to patients and their GPs. Br J Gen Pract.

[B29] Hickman M, Drummond N, Grimshaw J (1994). A taxonomy of shared care for chronic disease. J Public Health Med.

